# Commentary: Nationwide Surveillance of Novel Oxazolidinone Resistance Gene *optrA* in *Enterococcus* Isolates in China from 2004 to 2014

**DOI:** 10.3389/fmicb.2017.01631

**Published:** 2017-08-24

**Authors:** Gianluca Morroni, Andrea Brenciani, Serena Simoni, Carla Vignaroli, Marina Mingoia, Eleonora Giovanetti

**Affiliations:** ^1^Departments of Biomedical Sciences and Public Health, Polytechnic University of Marche Ancona, Italy; ^2^Departments of Life and Environmental Sciences, Polytechnic University of Marche Ancona, Italy

**Keywords:** *optrA* gene, OptrA protein, OptrA variants, enterococci, oxazolidinone resistance

A distictive feature of the novel oxazolidinone-phenicol resistance gene *optrA* is a variability yielding an encoded OptrA protein—a 655 amino acid sequence—which is variable in turn. The issue of the OptrA variants was more regularly addressed in the early studies of the new resistance than in the following reports. It is thus with particular interest that we read the recent nationwide surveillance study by Cui et al. (2016), where a wide screening of Chinese enterococci for *optrA* gave the authors an opportunity to repropose the issue of the different variants of the optrA protein.

When *optrA* was first reported in China from a 1998 to 2014 collection of human and animal enterococci (incidence, 2.0 and 15.9%, respectively), the *optrA*-carrying plasmid from a human *Enterococcus faecalis* isolate (E349) was sequenced (accession no. KP399637) (Wang et al., 2015). The relevant *optrA*-encoded protein is regarded as the wild type, and is hereinafter referred to as OptrA_E349_. Soon after the discovery of *optrA*, over a thousand enterococci, randomly collected in 2010–2014, were screened for the gene: among the *optrA*-positive isolates (incidence, 2.9%), nine different variants of the OptrA sequence (one being identical to OptrA_E349_) were detected (Cai et al., 2015). Seventeen *optrA*-positive, unrelated isolates of *E. faecalis* from the aforementioned 1998–2014 collection disclosed *optrA* sequences consistent with no new OptrA variant (seven isolates had OptrA_E349_) (He et al., 2016). Finally—in China, yet again—while screening over two thousand enterococci collected in 2004–2014, Cui et al. (2016) detected among the *optrA*-positive isolates (incidence, 2.0%) three new OptrA variants. Thus, the different OptrA sequences so far described in Chinese enterococci total 12 (including OptrA_E349_).

Meanwhile, as soon as the *optrA* sequence became available, we detected in Italy the gene—first report of *optrA* outside China—in two clinically distinct but virtually identical *Enterococcus faecium* isolates from a collection of 81 blood enterococci (incidence, 2.5%) recovered in 2015 (Brenciani et al., 2016). One of the two *E. faecium* isolates (strain E35048) was investigated for molecular traits, and its *optrA* gene (accession no. KT892063) displayed 98% DNA identity to the wild type gene.

In the light of the later data about the diversity of OptrA variants detected in China, it's apparent that our variant (hereinafter referred to as OptrA_E35048_) is much more dissimilar from OptrA_E349_ than Chinese variants. Altogether, the reported Chinese variants differ from OptrA_E349_ for two, three, or six amino acid substitutions, whereas OptrA_E35048_ differs from OptrA_E349_ for 21 substitutions, 17 of which (i.e., except those at positions 3, 12, 176, and 393) undetected in Chinese isolates. OptrA_E35048_ adds thus as a more distant variant to the OptrA variants detected in Chinese enterococci. OptrA_E349_ and the currently available enterococcal OptrA variants are summarized in Table 1 together with a number of relevant properties (the *optrA* gene location and the species, year of isolation, source, sequence type and linezolid MIC of individual isolates, whenever available). In particular, the frequent location of the *optrA* gene on conjugative plasmids makes the OptrA-mediated linezolid resistance transferable, an obvious cause for concern in view of possible resistance spread (Wang et al., 2015; He et al., 2016).

**Table 1:** *Enterococcus* isolates (plus one *Staphylococcus* isolate) where 14 different OptrA sequences (the *E. faecalis* wild type, 12 enterococcal variants, and 1 *S. sciuri* variant) have so far been documented.

**OptrA sequence**^**a**^		***optrA* gene location^b^**	**Isolates**					**References**
**Variant**	**Amino acid substitutions**		**Species^c^**	**Year of isolation**	**Source^d^**	**Sequence type**	**Linezolid MIC (μg/ml)**	
Wild type	−	p	*E. faecalis*	2009	h	116	8	Wang et al., 2015
(OptrA_E349_)		c	*E. faecalis*	2011	h	476	4	He et al., 2016
		nr^e^	*E. faecalis*	2012	h	476	8	Cai et al., 2015
		p	*E. faecalis*	2012	a	27	8	He et al., 2016
		c	*E. faecalis*	2012	a	619	8	He et al., 2016
		c	*E. faecalis*	2012	a	403	8	He et al., 2016
		nr	*E. faecalis*	2013	h	655	4	Cai et al., 2015
		nr	*E. faecalis*	2013	h	655	4	Cai et al., 2015
		nr	*E. faecalis*	2013	h	619	4	Cai et al., 2015
		nr	*E. faecalis*	2013	h	81	8	Cai et al., 2015
		nr	*E. faecalis*	2013	h	585	8	Cai et al., 2015
		nr	*E. faecalis*	2014	h	656	4	Cai et al., 2015
		nr	*E. faecalis*	2014	h	658	8	Cai et al., 2015
		p	*E. faecalis*	2014	h	585	16	He et al., 2016
		c	*E. faecalis*	2014	h	256	8	He et al., 2016
		p	*E. faecalis*	nr	h	480	16	He et al., 2016
		nr	8 *Enterococcus* sp.^f^	nr	h	nr	nr	Cui et al., 2016
RDK	Ile104Arg, Tyr176Asp, Glu256Lys	nr	*E. faecalis*	2012	h	207	8	Cai et al., 2015
		nr	*E. faecalis*	2014	h	314	8	Cai et al., 2015
		nr	4 *Enterococcus* sp.^f^	nr	h	nr	nr	Cui et al., 2016
DP	Tyr176Asp, Thr481Pro	nr	*E. faecalis*	2012	h	632	4	Cai et al., 2015
		nr	*E. faecalis*	2012	h	476	4	Cai et al., 2015
		nr	*E. faecalis*	2012	h	49	4	Cai et al., 2015
		p	*E. faecalis*	2012	a	59	4	He et al., 2016
		nr	*E. faecalis*	2013	h	16	4	Cai et al., 2015
		nr	*E. faecalis*	2013	h	480	8	Cai et al., 2015
		nr	*E. faecalis*	2013	h	480	8	Cai et al., 2015
		p	*E. faecalis*	2013	a	622	4	He et al., 2016
		nr	*E. faecalis*	2014	h	659	4	Cai et al., 2015
		p	*E. faecalis*	nr	h	480	4	He et al., 2016
		nr	7 *Enterococcus* sp.^f^	nr	h	nr	nr	Cui et al., 2016
EDM	Lys3Glu, Tyr176Asp, Ile622Met	nr	*E. faecalis*	2012	h	59	8	Cai et al., 2015
		nr	*E. faecalis*	2013	h	657	2	Cai et al., 2015
		nr	*E. faecalis*	2013	h	657	2	Cai et al., 2015
		c	*E. faecalis*	2013	h	16	4	He et al., 2016
		nr	*E. faecalis*	2014	h	591	2	Cai et al., 2015
		nr	*E. faecium*	2014	h	97	4	Cai et al., 2015
		nr	*E. thailandicus*	2014	h	nr	2	Cai et al., 2015
		nr	6 *Enterococcus* sp.^f^	nr	h	nr	nr	Cui et al., 2016
EDD	Lys3Glu, Tyr176Asp, Gly393Asp	c	*E. faecalis*	2012	a	93	2	He et al., 2016
		nr	*E. faecalis*	2013	h	192	4	Cai et al., 2015
		nr	*E. gallinarum*	2014	h	nr	2	Cai et al., 2015
		nr	6 *Enterococcus* sp.^f^	nr	h	nr	nr	Cui et al., 2016
KD	Thr112Lys, Tyr176Asp	p	*E. faecalis*	2012	a	116	8	He et al., 2016
		nr	*E. faecalis*	2013	h	16	8	Cai et al., 2015
		nr	*E. faecalis*	2013	h	16	8	Cai et al., 2015
		p	*E. faecalis*	2013	a	330	8	He et al., 2016
		nr	*E. faecalis*	2014	h	16	8	Cai et al., 2015
		p	*E. faecalis*	nr	h	480	2	He et al., 2016
		nr	3 *Enterococcus* sp.^f^	nr	h	nr	nr	Cui et al., 2016
EYDNDM	Lys3Glu, Asn12Tyr, Tyr176Asp, Asp247Asn, Gly393Asp, Ile622Met	nr	*E. faecalis*	2010	h	593	2	Cai et al., 2015
		nr	*E. faecalis*	2014	h	368	2	Cai et al., 2015
		nr	*E. faecalis*	2014	h	593	2	Cai et al., 2015
		nr	1 *Enterococcus* sp. ^f^	nr	h	nr	nr	Cui et al., 2016
EDP	Lys3Glu, Tyr176Asp, Thr481Pro	nr	*E. faecalis*	2014	h	480	4	Cai et al., 2015
DD	Tyr176Asp, Gly393Asp	c	*E. faecalis*	2009	a	21	2	He et al., 2016
		nr	*E. faecium*	2011	h	885	4	Cai et al., 2015
		c	*E. faecalis*	2013	h	27	8	He et al., 2016
		nr	*E. faecium*	2010	h	882	4	Cai et al., 2015
		nr	2 *Enterococcus* sp.^f^	nr	h	nr	nr	Cui et al., 2016
DK	Tyr176Asp, Glu256Lys	nr	1 *Enterococcus* sp.^f^	nr	h	nr	nr	Cui et al., 2016
ED	Lys3Glu, Tyr176Asp	nr	3 *Enterococcus* sp.^f^	nr	h	nr	nr	Cui et al., 2016
KDP	Thr112Lys, Tyr176Asp, Thr481Pro	nr	4 *Enterococcus* sp.^f^	nr	h	nr	nr	Cui et al., 2016
LEYYWDV DASKELY NKQLEIG (OptrA_E35048_)	Met1Leu, Lys3Glu, Asn12Tyr, Asn122Tyr, Tyr135Trp, Tyr176Asp, Ala350Val, Gly393Asp, Val395Ala,Ala396Ser, Gln509Lys, Gln541Glu, Met542Leu, Asn560Tyr, Lys562Asn, Gln565Lys, Glu614Gln, Ile627Leu, Asp633Glu, Asn640Ile, Arg650Gly	nr	*E. faecium*	2015	h	117	4	Brenciani et al., 2016
EYDD	Lys3Glu, Asn12Tyr, Tyr176Asp, Gly393Asp	p	*S. sciuri*	2013	a	nr	16	Li et al., 2016

a *Variant: the substituting amino acids are given using the single-letter code. Substitutions: amino acid substitutions and their positions*.

b *optrA location: p, plasmid; c, chromosome*.

c *All species reported in this column are Enterococcus species, except for the one reported on the last line which is a Staphylococcus species (S. sciuri)*.

d *Source: h, human; a, animal*.

e *nr, not reported*.

f *Enterococcus species not specified*.

While recently investigating three *optrA*-positive *E. faecalis* isolates of poultry origin in Colombia, Cavaco et al. (2017) deduced that two carried an *optrA* gene identical to one already detected in China, whereas the third isolate bore an *optrA* gene with a different nucleotide sequence that was defined as “more closely related” to the one we had described in Italy.

Worryingly, the *optrA* gene has been found in China not only in enterococci, but also in staphylococci, specifically in a *Staphylococcus sciuri* strain of swine origin (Li et al., 2016): *optrA* and its promoter region exhibited 99.1% nucleotide sequence identity to the corresponding region on the wild type *E. faecalis* plasmid pE349. The 655 amino acid OptrA sequence from *S. sciuri* is another variant exhibiting 99.4% identity to OptrA_E349_ (Table 1).

It's self-evident that *optrA* is not a conserved gene. The related variability of OptrA proteins appears to be a fitting example of that evolvability of clinical resistance by the antibiotic's effect which has been the subject of a recent reflection by Baquero et al. (2013). Given the importance of oxazolidinones as last resort antibiotics for the treatment of serious infections caused by Gram-positive pathogens, it would be important to clarify how the different amino acid substitutions affect OptrA-mediated resistance. However, irrespective of the variant, the linezolid MICs for the *optrA*-positive enterococci listed in Table 1 display limited variability (2–16 μg/ml), the highest MIC value in the range being shared by the *optrA*-positive strain of *S. sciuri*. Remarkably, our *optrA*-positive *E. faecium* (Brenciani et al., 2016), in spite of no less than 21 amino acid substitutions, exhibits the same linezolid MIC (4 μg/ml) as several Chinese isolates with other OptrA variants, suggesting that the number of amino acid substitutions has little influence on the level of linezolid resistance.

On the other hand, the linezolid resistance breakpoint is still an unsettled issue: indeed, an enterococcus with a linezolid MIC of 4 μg/ml is regarded as “intermediate” according to Clinical Laboratory Standards Institute (2017) and “susceptible” according to European Committee on Antimicrobial Susceptibility Testing (2017). The latter Committee, in particular, sets for enterococci a linezolid epidemiological cut-off of 4 μg/ml, and has increased the susceptible clinical breakpoint of linezolid to ≤4 μg/ml to avoid dividing wild type MIC distributions (European Committee on Antimicrobial Susceptibility Testing, 2017). In spite of the low linezolid MICs for several *optrA*-positive isolates, it's well established that in the clinical setting, as well as with other antibiotics, resistance levels may increase in patients with risk factors such as previous linezolid therapy, prolonged exposure to linezolid, and intensive care unit stay (Endimiani et al., 2011; Mendes et al., 2014).

In conclusion, we share and support many recent studies recommending routine surveillance of enterococci for the presence of the *optrA* gene. In addition, however, we wish for a more extensive interest in the OptrA variants and their correlation with oxazolidinone and phenicol MICs.

## Author contributions

All authors listed have made a substantial, direct and intellectual contribution to the work, and approved it for publication.

### Conflict of interest statement

The authors declare that the research was conducted in the absence of any commercial or financial relationships that could be construed as a potential conflict of interest.

## References

[B1] Baquero F. Tedim A. P. Coque T. M. (2013). Antibiotic resistance shaping multi-level population biology of bacteria. Front. Microbiol. 4:15. 10.3389/fmicb.2013.00015 23508522 PMC3589745

[B2] Brenciani A. Morroni G. Vincenzi C. Manso E. Mingoia M. Giovanetti E.. (2016). Detection in Italy of two clinical *Enterococcus faecium* isolates carrying both the oxazolidinone and phenicol resistance gene *optrA* and a silent multiresistance gene *cfr*. J. Antimicrob. Chemother. 71, 1118–1119. 10.1093/jac/dkv438 26702919

[B3] Cai J. Wang Y. Schwarz S. Lv H. Li Y. Liao K.. (2015). Enterococcal isolates carrying the novel oxazolidinone resistance gene *optrA* from hospitals in Zhejiang, Guangdong, and Henan, China, 2010-2014. Clin. Microbiol. Infect. 21, 1095.e1–1095.e4. 10.1016/j.cmi.2015.08.007 26319902

[B4] Cavaco L. M. Bernal J. M. Zankari E. Léon M. Hendriksen R. S. Perez-Gutierrez E.. (2017). Detection of linezolid resistance due to the *optrA* gene in *Enterococcus faecalis* from poultry meat from the American continent (Colombia). J. Antimicrob. Chemother. 72, 678–683. 10.1093/jac/dkw490 27999039

[B5] Clinical and Laboratory Standards Institute (2017). M100-S27. Performance Standards for Antimicrobial Susceptibility Testing; Twenty-Seventh Informational Supplement, Wayne, PA: Clinical and Laboratory Standards Institute.

[B6] Cui L. Wang Y. Lv Y. Wang S. Song Y. Li Y.. (2016). Nationwide surveillance of novel oxazolidinone resistance gene *optrA* in *Enterococcus* isolates in China from 2004 to 2014. Antimicrob. Agents Chemother. 60, 7490–7493. 10.1128/AAC.01256-16 27645239 PMC5119033

[B7] Endimiani A. Blackford M. Dasenbrook E. C. Reed M. D. Bajaksouszian S. Hujer A. M.. (2011). Emergence of linezolid-resistant *Staphylococcus aureus* after prolonged treatment of cystic fibrosis patients in Cleveland, Ohio. Antimicrob. Agents Chemother. 55, 1684–1692. 10.1128/AAC.01308-10 21263048 PMC3067150

[B8] European Committee on Antimicrobial Susceptibility Testing (2017). Available online at: http://www.eucast.org/

[B9] He T. Shen Y. Schwarz S. Cai J. Lv Y. Li J.. (2016). Genetic environment of the transferable oxazolidinone/phenicol resistance gene *optrA* in *Enterococcus faecalis* isolates of human and animal origin. J. Antimicrob. Chemother. 71, 1466–1473. 10.1093/jac/dkw016 26903276

[B10] Li D. Wang Y. Schwarz S. Cai J. Fan R. Li J.. (2016). Co-location of the oxazolidinone resistance genes *optrA* and *cfr* on a multiresistance plasmid from *Staphylococcus sciuri*. J. Antimicrob. Chemother. 71, 1474–1478. 10.1093/jac/dkw040 26953332

[B11] Mendes R. E. Deshpande L. M. Jones R. N. (2014). Linezolid update: stable *in vitro* activity following more than a decade of clinical use and summary of associated resistance mechanisms. Drug Resist. Updat. 17, 1–12. 10.1016/j.drup.2014.04.002 24880801

[B12] Wang Y. Lv Y. Cai J. Schwarz S. Cui L. Hu Z.. (2015). A novel gene, *optrA*, that confers transferable resistance to oxazolidinones and phenicols and its presence in *Enterococcus faecalis* and *Enterococcus faecium* of human and animal origin. J. Antimicrob. Chemother. 70, 2182–2190. 10.1093/jac/dkv116 25977397

